# Diagnosing *Rickettsia felis* infection with Metagenomic Next-Generation Sequencing (mNGS) in a patient with ankylosing spondylitis: a case report and literature review

**DOI:** 10.3389/fimmu.2025.1643599

**Published:** 2025-09-11

**Authors:** Mengyao Liu, Yang Cheng, Xinchang Wang, Zhaochun He, Kepeng Yang, Kai Chen, Yongsheng Fan, Weijie Wang

**Affiliations:** ^1^ The Second Clinical Medical College of Zhejiang Chinese Medical University, Hangzhou, China; ^2^ The Second Affiliated Hospital of Zhejiang Chinese Medical University, Hangzhou, China

**Keywords:** ankylosing spondylitis, mNGS, Rickettsia felis, fever of unknown origin, literature review, case report

## Abstract

*Rickettsia felis*, an emerging flea-borne pathogen with global distribution potential, is a neglected cause of undifferentiated febrile illness, although reported human cases remain sparse. The development of molecular diagnostic methods, along with the application of metagenomic next-generation sequencing (mNGS), has improved the diagnostic accuracy of infectious fevers. A case of *Rickettsia felis* infection was diagnosed by mNGS in a 55-year-old patient with pre-existing ankylosing spondylitis. Five previously reported cases of *Rickettsia felis* infection were systematically reviewed, with a comprehensive analysis of their epidemiological characteristics, clinical manifestations, and therapeutic regimens. This study highlights the clinical features and diagnostic approaches of the disease through a case report and literature review.

## 1 Introduction

Among critically ill patients, infections are a leading cause of death. In recent years, the incidence and mortality rates of infections have remained high due to the emergence of new pathogens, the proliferation of drug-resistant strains, and the increasing population of immunosuppressed individuals. Severe infections exhibit a rapid onset and progression and involve a diverse array of pathogens. Rapid identification of causative microorganisms is therefore crucial.

Over the past two decades, advancements in high-throughput sequencing and computational biology have led to the development of transformative diagnostic tools, enabling significant progress in infection detection. Metagenomic Next-Generation Sequencing (mNGS) is a high-throughput technology capable of simultaneously processing numerous DNA/RNA fragments from blood and cerebrospinal fluid (CSF) samples, generating extensive genomic datasets for comprehensive analysis of pathogen species and genetic information within samples (1, 2). mNGS does not rely on traditional microbial cultures, allowing rapid and unbiased testing of clinical samples for a wide range of pathogenic microorganisms without the need for specific amplification. It is particularly suitable for the critical and challenging diagnosis of infections. Molecular diagnostic approaches, including polymerase chain reaction (PCR) and mNGS, enable earlier pathogen detection compared to conventional culture-based methods. PCR is a targeted technique used to detect specific gene sequences of known pathogens. In contrast, the unbiased detection capability of mNGS allows it to identify a broader range of unknown pathogens. For fever of unknown origin or infectious diseases, mNGS can serve as a comprehensive screening tool to help identify potential novel pathogens or mixed infections. Using mNGS, neurologists and other subspecialists in internal medicine, infectious diseases, critical care, and rheumatology can determine whether a latent infection exists (3). One study demonstrated that mNGS of bronchoalveolar lavage fluid (BALF) efficiently identifies causative pathogens in pediatric pneumonia (4). Another research team developed a cell-free DNA-based mNGS assay for body fluids, which demonstrated superior sensitivity and specificity compared to microbiological testing using culture and PCR in their comparative analysis (5).

This case report describes a 55-year-old female patient with *Rickettsia felis* (*R. felis*) infection, highlighting the clinical utility of mNGS in identifying the etiology of her febrile illness.

## 2 Materials and methods

mNGS of patient serum samples in this study was performed using the “RealPathogen” platform. This platform analyzes both microbial cellular nucleic acids and cell-free DNA/RNA fragments, employing high-throughput sequencing on Illumina NextSeq 550 systems. Pathogen identification was achieved by alignment with a curated microbial reference database, which includes 9,757 bacterial species (excluding 169 Mycobacteria and 126 Mycoplasma/Chlamydia species), 6,874 viruses, 1,563 fungi, and 297 parasites with known genomic sequences, followed by proprietary bioinformatic algorithms. The standard reporting time was 24–48 h for DNA detection and 48–72 h for RNA analysis. Further inquiries may be directed to the corresponding author.

## 3 Case presentation

### 3.1 Clinical presentation and physical examination

A 55-year-old Chinese woman with a 7-year history of ankylosing spondylitis (AS), treated with methotrexate 10 mg weekly and tofacitinib 5 mg twice daily, was admitted to the Second Affiliated Hospital of Zhejiang Chinese Medical University (Hangzhou, Zhejiang Province, China) on 14 August 2020, presenting with afternoon fever. Since her AS diagnosis, she has been regularly followed at our hospital every 3 months.

Her medical history included vulvar condyloma acuminatum and intraepithelial neoplasia, with Human papillomavirus (HPV)39 and HPV16 positivity for the past 2 years. Recombinant human interferon thrombolytic agents were prescribed for vaginal administration every other day. The patient served as the Dean of the Elderly Welfare Institute, a role that involved a stressful and demanding lifestyle while managing daily affairs. She has never traveled outside of China and resides in Hangzhou, Zhejiang Province. She was a widow living with a cat and reported no recreational drug use. She did not smoke, drink alcohol, or use illicit drugs.

### 3.2 Clinical findings

Upon physical examination, the patient had a fever ranging from 38°C to 39°C. While breathing ambient air, her heart rate was 77 beats per minute, blood pressure was 135/77 mmHg, respiratory rate was 18 breaths per minute, and oxygen saturation was 99%. There was limited lateral bending and extension of the waist, a positive finger-to-ground test, a “four” word test, and a straight leg raising test. No obvious tenderness was observed in the back of the waist or sternum, and there was no swelling, pain, or deformity in other peripheral joints. There were no lesions in the oropharynx, and her neck was supple. The lungs were clear, and her heart rhythm was regular, without murmurs. The abdomen was not tender, and no organomegaly was noted. The neurological examination was unremarkable.

Laboratory examinations showed positivity for HLA-B27 (4,578.00). Antinuclear antibody testing revealed no abnormalities. Initial laboratory evaluation revealed a white blood cell (WBC) count of 2.7 × 10^9^/L, with 55.7% neutrophils and 29.7% lymphocytes. The red blood cell (RBC) count was 3.57 × 10^12^/L, hemoglobin was 97 g/L, and platelets were 147 × 10^9^/L. C-reactive protein (CRP) was 7.65 mg/L. Liver and kidney function test results were normal. Increased IgG and erythrocyte sedimentation rate (ESR) levels were noted. Urinalysis revealed BLD + − (10) RBC/μl, LEU + (70) WBC/μl, AVBXB 16/μl, and AVSP 29/μl (**Table 1**). Chest CT showed proliferative foci in the lower left lobe of the lung, with thickening and calcification of the right pleura.

**Table 1:** Laboratory tests during hospitalization.

Test	Results at admission (8.13)	Results on Day 2 of fever (8.18)	Results at 8.28	Results at 8.30	Normal value
WBC (× 10^9^/L)	2.7	2.5	4.3	3.9	3.5–9.5
RBC (× 10^12^/L)	3.57	3.36	3.48	3.37	3.80–5.10
HGB (g/L)	104	97	99	97	115–150
PLT (× 10^9^/L)	147	111	164	130	125–350
CRP (mg/L)	7.65	6.60	2.11	7.56	0–10
ESR (mm/h)	48	–	41	49	0–20
ALT (U/L)	17	–	16	–	7–40
AST (U/L)	35	–	33	–	13–35
CREA (μmol/L)	55.4	–	56.6	–	41.0–81.0
IgG (g/L)	17	–	13	–	7.51–15.60
BLD (RBC/μl)	± (10)	–	–	–	Negative
LEU (WBC/μl)	+ (70)	–	–	–	Negative

WBC, white blood cell; RBC, red blood cell; HGB, hemoglobin; PLT, platelet count; CRP, C-reactive protein; ESR, erythrocyte sedimentation rate; ALT, alanine aminotransferase; AST, aspartate aminotransferase; CREA, creatinine; IgG, immunoglobulin G; BLD, blood; LEU, leucocyte.

### 3.3 The progression of disease treatment

**Figure 1:**
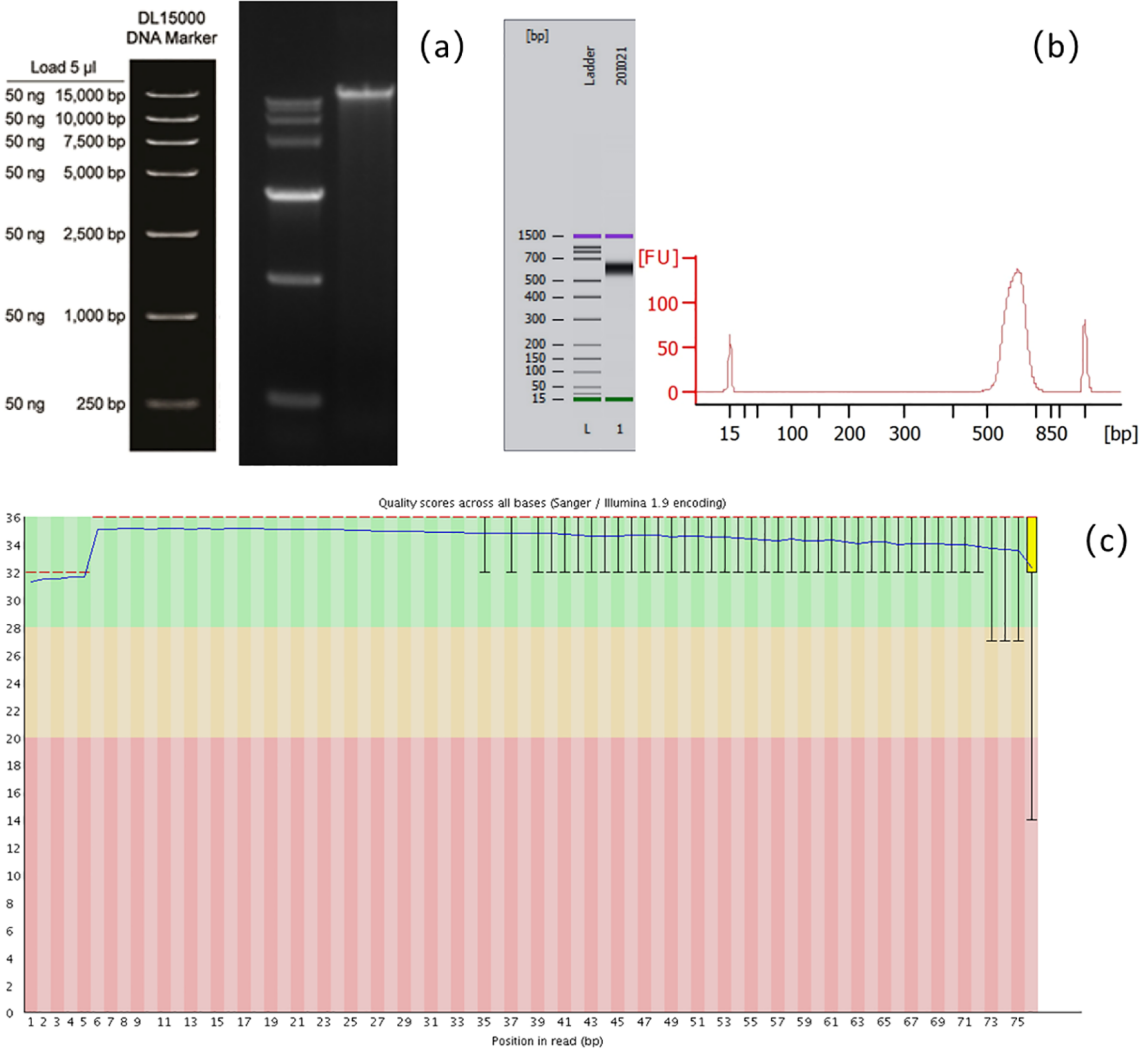
Schematic diagram of quality control of the mNGS assay. **(a)** Agarose gel electrophoresis for detecting the integrity of nucleic acid fragments. **(b)** Agilent 2100 Bioanalyzer for detecting the size of library fragments. **(c)** Base quality detection of sequencing reads.

**Figure 2:**
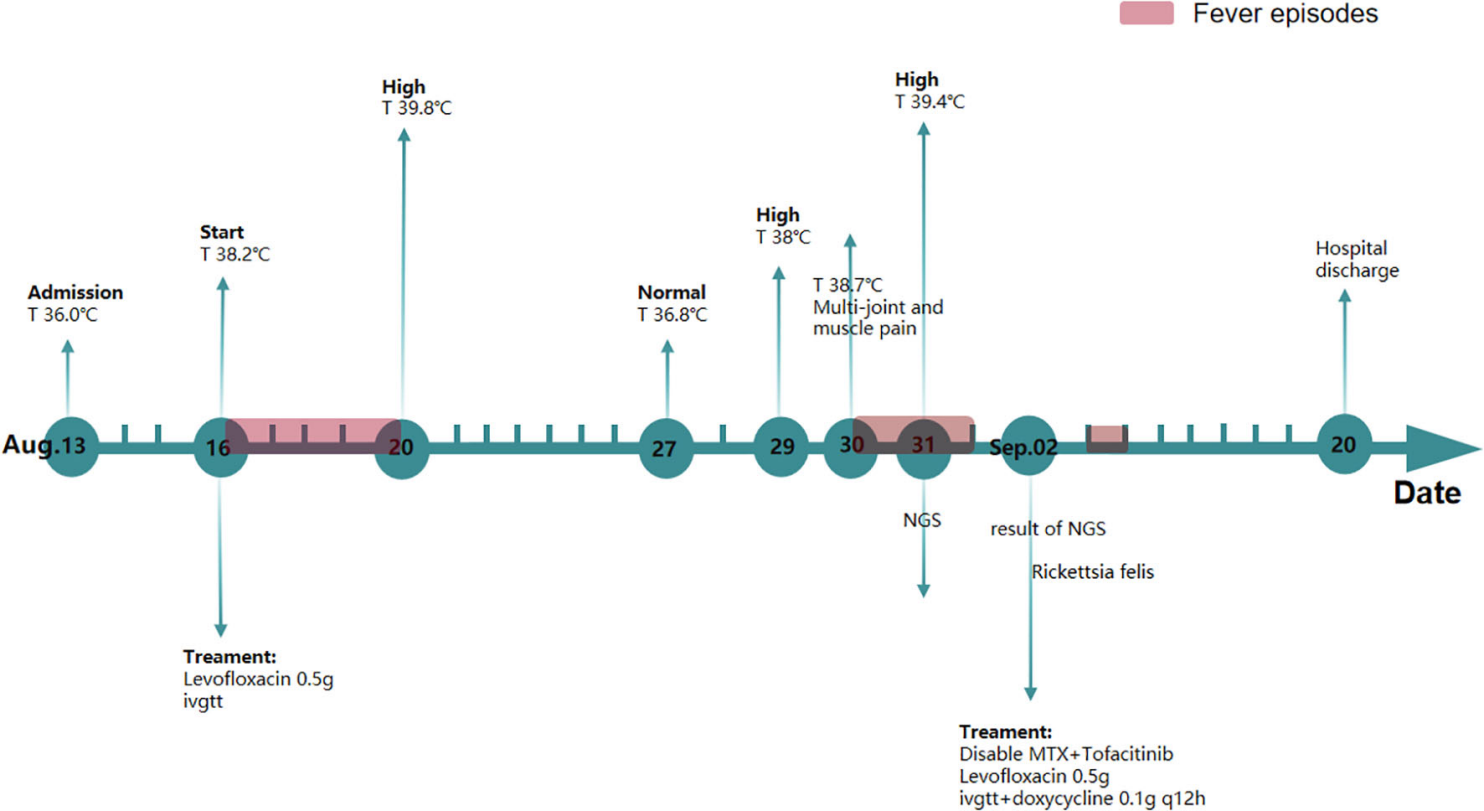
Changes in temperature, symptoms, and medication during the patient’s hospital admission.

Upon hospitalization, CRP levels were normal, while ESR levels were elevated. The patient also exhibited limited waist and back mobility. The medical team speculated that she was in the active phase of AS. The treatment plan included methotrexate 10 mg once weekly, tofacitinib 5 mg once daily, and loxoprofen 5 mg as needed for elevated body temperature. Over the following days, despite undergoing treatment, the patient experienced recurring episodes of high fever. Swab, urine, blood cultures, TB-IGRA (tuberculosis), and fungal cultures were negative. A re-examination of the TCT report on 26 August 2020 showed low-grade squamous intraepithelial lesions. Human papillomavirus genotyping revealed positivity for HPV39 and HPV16. Urinalysis indicated a urinary tract infection, although the patient reported no symptoms such as frequent urination, urgency, or dysuria. Consequently, the gynecologist recommended one recombinant human interferon suppository every other day and levofloxacin 0.5g daily via vaginal administration. The patient did not respond to presumptive treatment. Based on this, the medical team hypothesized the presence of rare pathogens. Testing identified the pathogen as *R. felis*, a Gram-negative bacterium with sequence number 62 at the species level and a relative abundance of 1.11%, and sequence number 73 at the genus level (**Figure 1**). Therefore, doxycycline (0.1 g/12h) was administered orally for infection control. Subsequently, the patient’s temperature returned to normal without further fluctuations. The WBC count was 3.9 × 10^9^/L, and CRP was 7.56 mg/L, both within the normal range. A 16-day course of doxycycline resulted in an uneventful recovery without fever recurrence (**Figure 2**). After 3 months of follow-up, the patient remained afebrile.

## 4 Discussion

To date, no cases of AS infection by *R. felis* have been reported in humans. The patient’s fever of unknown origin (FUO) resolved following appropriate treatment. *R. felis* is an obligate intracellular alpha-proteobacterium with broad ecological versatility, capable of infecting diverse vectors and hosts. The cat flea (*Ctenocephalides felis*) is recognized as its primary transmission vector and reservoir host (6, 7). Accumulating evidence also confirms its presence in other arthropods, including ticks, mites, lice, and mosquitoes. Concurrently, the host range of *R. felis* continues to expand, with confirmed infections reported globally in humans, domestic and wild small mammals, and other species (8). It was first discovered in the USA in 1990, with the first human case reported there in 1994 (9). Since then, hundreds of cases have been documented across more than 20 countries on five continents. This disease has no obvious regional preference and has the potential for global transmission (10). In China, cases of *R. felis* infection have been reported in multiple provinces. The initial detection via PCR occurred in Jiangsu Province in 2014 (8), followed by subsequent reports in Shandong, Guangdong, Taiwan, and other regions. Clinical manifestations of *R. felis* infection include central nervous system and respiratory involvement. A retrospective study in China reported that rickettsial infections accounted for 2.14% of patients with unexplained fever (11), although mortality data specific to *R. felis* are not available.

Studies have shown that host factors significantly influence the severity of rickettsial infections. Significant risk factors include advanced age, glucose-6-phosphate dehydrogenase (G6PD) deficiency, diabetes mellitus, and male sex (12). The risk of *R. felis* infection is also elevated by several behavioral factors, including ownership of domestic animals (particularly absence of routine veterinary surveillance in free-roaming animals), exposure to *Ctenocephalides felis* vectors (whether through pet contact or environmental exposure, further increases transmission likelihood), and outdoor activities such as hiking in forested endemic areas, which increase contact with infected arthropod vectors. The main symptoms of *R. felis* infection include fever, pain, and rash or eschar-based skin manifestations. In some cases, patients may also present with elevated liver enzymes and other indicators of liver and kidney dysfunction (13). A comprehensive literature search was conducted across multiple databases, including PubMed, Embase, Web of Science, and the Cochrane Library, without language restrictions. The search strategy employed the following key terms: *Rickettsia felis*, *Rickettsiales*, *Rickettsiaceae*, *Rickettsieae*, *R. felis*, and *Rickettsia.* The search was restricted to “case reports” published from January 2014 to July 2025. **Table 2** presents the clinical characteristics of eight patients in eight studies. The cohort included five men and five women, with a mean age of 34.8 years. Fever was absent as the first symptom in only one patient, while six patients experienced headaches. Six patients reported a history of contact with dogs and cats, and three had a history of arthropod bites. Most CSF or blood samples were tested for trichinosis using mNGS. Clinical improvement was observed in most patients after 5–14 days of treatment. Given the wide range of reported symptoms, some studies suggest that clinical manifestations may be influenced by the patient’s geographic location (13). Patients from tropical regions typically present with fever, while those from Europe or the Americas often exhibit fever accompanied by skin manifestations. In parts of Asia, such as Thailand and South Korea, symptoms may include fever along with myalgia, chest pain, and joint pain. *R. felis* infections are generally responsive to doxycycline; however, diagnosis remains challenging. Inadequate or inappropriate treatment can result in severe complications, including hepatomegaly, myocarditis, meningoencephalitis, and other organ involvement, potentially leading to poor prognosis or even death.

**Table 2:** Clinical features of *Rickettsia felis* infection reported in the literature.

Case/year	Country	Age/sex	Symptoms	Modes of transmission to humans	Underlying disease	Diagnosis method	Sample	Treatment	Duration of treatment
1/2024 (16)	China	26/F	Light coma	Long-term employment in a garment factory and exposure to duck-down feathers	NA	NGS	Blood and CSF	Azithromycin	17 days
2/2024 (17)	China	43/M	Persistent cough and fever	Ingested raw freshwater fish and residential exposure to cats/dogs	A history of *Clonorchis sinensis* and recent corticosteroid use	mNGS	Blood and BALF	Doxycycline	NA
3/2023 (18)	China	23/F	Headache, fever, weakness in both lower limbs	Cat ownership	A history of thrombocytopenia	mNGS	CSF	Empirical antituberculosis treatment (rifampicin) combined with a steroid	10 days
		29/M	Headache, fever	NA	NA	mNGS	CSF	Doxycycline	14 days
4/2022 (19)	Guatemala	3/M	Fever, cough, dyspnea, vomiting, abdominal pain, weakness	Cat ownership	NA	PCR	Blood sample	NA	NA
5/2022 (20)	China	47/M	Fever, myalgia	NA	Chronic kidney disease	mNGS	Blood sample	Doxycycline plus moxifloxacin	NA
6/2015 (21)	Italian	57/F	Fever, headache, nausea, vomiting, rash	Traveled to Nepal and was attacked by aquatic leeches	NA	Indirect immunofluorescence	Blood sample	Oral doxycycline and intravenous ceftriaxone	14 days + 10 days
7/2014 (22)	Thailand	20/F	Fever, myalgia, arthralgia, headache, abdominal pain, cough, chest pain	Dogs, bitten by insects and mosquitoes	NA	Immunofluorescent	Serum samples	NA	NA
		45/M	Fever, myalgia, arthralgia, headache, cough, abdominal pain, chest pain, vomiting, photophobia	Cats, dogs, bitten by insects or mosquitoes	NA	Immunofluorescent	Serum samples	NA	NA
8/this case	China	55/F	Fever	Cat ownership	NA	mNGS	Blood sample	Doxycycline	16 days

CSF, cerebrospinal fluid; BALF, bronchoalveolar lavage fluid.

AS is a chronic inflammatory disorder characterized by inflammation of the vertebral and sacroiliac joints (14), often with an insidious early onset. Fever in AS patients typically indicates inflammatory activity and is frequently accompanied by elevated CRP, ESR, and local or systemic inflammatory responses. Infection is an important contributor to the pathogenesis of AS and can influence disease progression. In the present study, most infectious foci in AS patients were located in the respiratory tract, gastrointestinal tract, genitourinary tract, and eyes, manifesting as pneumonia, inflammatory bowel disease, herpes zoster, and uveitis. Elevated body temperature in AS patients—or in otherwise healthy individuals without a significant increase in inflammatory markers—poses a diagnostic challenge for physicians. In this case, the patient lived with a cat and presented after admission with symptoms consistent with rickettsial infection, including persistent fever and systemic pain. She had a history of AS for more than 7 years and long-term use of immunosuppressive drugs. Additionally, she had a history of cervical intraepithelial neoplasia and vulvar condyloma acuminatum. These factors are likely to affect the patient’s immune function, which may explain why her temperature continued to fluctuate significantly despite levofloxacin treatment. Autoimmune diseases, including AS, increase the likelihood of secondary infections due to the use of medications such as hormones and immunosuppressants, making it crucial to identify the underlying cause of infection. mNGS, as a culture-independent diagnostic method, plays an important role in clarifying the etiology of such infections (1). Here, mNGS of the patient’s serum sample identified infection with *R. felis*, enabling the medical team to formulate an individualized and targeted treatment plan. Following doxycycline therapy against *R. felis*, the patient’s temperature returned to normal, with no further abnormal fluctuations. Therefore, when broad-spectrum antibiotic therapy is not effective, clinicians should consider the possibility of atypical pathogen infections and implement corresponding individualized treatment strategies to prevent disease progression.

At present, information on the clinical manifestations of *R. felis* infection remains limited. It is difficult to determine whether the patient’s asymptomatic urinary tract infection is specifically related to cat-associated rickettsia, beyond her age and medical history. Although our understanding of *R. felis* has advanced considerably in recent years, many aspects of its ecology and epidemiology remain unclear. For patients with autoimmune diseases, it is important to minimize exposure to potential sources of infection, such as reducing contact with cats and dogs and maintaining good environmental hygiene. For clinicians, mNGS can substantially improve the early diagnosis of infectious diseases due to its fundamental advantages, including culture-independent, unbiased pathogen detection directly from clinical specimens and a rapid turnaround time (typically 24–48 h) (15). However, the clinical adoption of mNGS remains limited by its high cost, as it is typically offered as a laboratory-developed test (LDT) by specialized clinical or commercial laboratories. Rather than replacing conventional diagnostic methods, mNGS should be regarded as a complementary tool. This limitation continues to be actively investigated, and further evidence is required to establish standardized clinical implementation guidelines for mNGS (2). From a public health perspective, the identification of human infections in individuals presenting with FUO or rash, particularly those with a history of animal contact, necessitates ongoing surveillance and investigation of indigenous reservoirs and vectors of *R. felis* within the community.

## Data Availability

The datasets generated and analyzed during the current study are available in the SRA repository with accession number: PRJNA1148913. Further inquiries can be directed to the corresponding author.
